# The feasibility and acceptability of digital technology for health and wellbeing in social housing residents in Cornwall: A qualitative scoping study

**DOI:** 10.1177/20552076221074124

**Published:** 2022-01-24

**Authors:** Sarah Ann Buckingham, Tim Walker, Karyn Morrissey, on behalf of the Smartline project team

**Affiliations:** 1 European Centre for Environment and Human Health, Royal Cornwall Hospitals NHS Trust, 151778 University of Exeter Medical School, Truro, UK; 2 Centre for Geography and Environmental Science, 151778 University of Exeter, Penryn, UK; 3 Sustainability Division, Department of Technology, 151778 Management and Economics, Technical University of Denmark

**Keywords:** Digital, general health, general wellbeing, psychology, social housing, qualitative studies

## Abstract

**Objective:**

The aim of this study was to explore the feasibility and acceptability of digital technology for improving health and wellbeing in social housing residents living in a deprived area in Cornwall, England.

**Methods:**

Qualitative scoping study with focus groups and telephone interviews (23 participants in total). Focus groups and interviews were audio-recorded, transcribed verbatim and analysed thematically.

**Results:**

Levels of use and experience with digital technology were diverse in this group, ranging from ‘willing and unable’ to ‘expert’ on a self-perceived scale. Overall, participants had positive perceptions of technology and were keen to try new technologies. Five categories of factors influencing technology use were identified: functional, physical / health, psychological and attitudinal, technology-associated barriers, and privacy, safety and security. Preferred types of digital technology were wearable activity monitors (e.g. Fitbit®), virtual assistants (e.g. Amazon Alexa) and social messaging (e.g. WhatsApp). There was a strong consensus that technology should be easy to use and should have a clear purpose. There was a need to improve awareness, knowledge and confidence in technology use and participants desired further training and support.

**Conclusions:**

There is a need and desire to use digital technology to improve health, wellbeing and social connectedness in social housing residents in Cornwall. The findings will be used to inform a digital training and support programme for the participants of the Smartline project. This study also serves as a template for future research that seeks to scope the feasibility and acceptability of different digital interventions in similar populations.

## Introduction

Digital technology (such as smartphone applications, wearable activity monitors, virtual assistants, social networking and video calling software) provides an appealing, accessible, scalable and low cost tool to promote health and wellbeing.^1^ Potential benefits include the promotion of positive health behaviour change, for example increasing physical activity and reducing sedentary behaviour,^1–3^ and reducing social isolation and feelings of loneliness.^4,5^ Although there is some evidence to support the acceptability and impact of various digital technologies for health and wellbeing, this connection is far from established. Reviews have reported existing studies to be short-term, limited in scope and sample size, and with a high risk of bias.^2,4,6,7^ There also remains an emphasis on quantitative measures of effectiveness, often at the expense of qualitative methods that capture important aspects such as feasibility, acceptability and engagement.^8^

With technology underpinning modern economic and social activity, the consequences of digital exclusion are pervasive and will impact people's access to labour markets, services, and social and civic participation.^9^ The definition of digital exclusion has changed over the years from a simple ‘user/non-user’ perspective, to include different levels of Information and Communications Technology (ICT) and internet use and skills divisions.^10^ The concept of digital competence goes a step further by recognising that digital exclusion is not only affected by skills but also knowledge, cognition, awareness, attitudes, and ethics.^11^ Competency levels are determined by how these are drawn on and mobilised together in order to meet complex digital tasks.^12^ Low levels of digital competence among different groups of the population mean that many remain digitally excluded. Social housing residents are a unique population that is likely to have much to gain from access to and use of digital technology,^9^ but may be disproportionately affected by digital exclusion. This group tends to have many of the characteristics associated with digital exclusion, including older age, high unemployment, low income, low education and low social mobility.^13^ Those living in rural areas are also more likely to be digitally excluded.^14^

Working with a unique cohort of 263 households in social housing in a geographically isolated area in the UK, the Smartline project and the Smartline Extension project are a six-year research programme funded by the European Regional Development Fund.^15–17^ The overarching aim of the project is to explore and trial the opportunities for technology to support people to live healthier and happier lives in their homes and communities.^15^ Evidence suggests that involving participants in the design and implementation of interventions is important.^18^ Studies that engage users at various stages of the project may be more successful in achieving the desired outcomes than those that do not.^19,20^ Within this context, a user-centred approach to understanding the feasibility and acceptability of digital technology for health and wellbeing is a core research theme within the overall Smartline project.

Data collected from a 2019 survey with the social housing community involved in the Smartline project revealed low levels of digital access and engagement. Of 483 respondents, 101 (21%) did not have access to the internet.^21^ Those that responded to a supplementary question explained that the main reason for lack of access is that they do not wish to use the internet. Other reasons cited were affordability and a need for support or training.

Within this context, this scoping study involved the use of qualitative methods to explore the feasibility and acceptability of digital technology for improving health and wellbeing in the Smartline social housing cohort, in order to inform subsequent interventions. The aims were: to explore existing digital technology use and competence; to explore digital willingness and readiness to use new technologies; to identify perceived barriers and facilitators to technology use; and to scope the feasibility and acceptability of potential digital interventions.

## Methods

### Research design

A descriptive qualitative design was used for this study, with focus group and interview methods. An interpretivist paradigm was seen as most appropriate given the nature of the research questions, the type of data collected (i.e. mainly qualitative), and the need to gain an understanding of individual experience from the perspective of the person experiencing it. This approach recognises the importance of subjective interpretation, perceptions and meaning attached to experiences.^22^

### Study setting and participants

Three focus groups (*n* = 19) of approximately 90 min duration were conducted in February 2020. Each focus group included between five and nine participants. Focus groups took place in meeting spaces (such as café meeting rooms) in Camborne, Pool and Redruth, with the locations selected based on convenience for the participants. One-to-one telephone interviews (*n* = 4) were conducted following the focus groups; the duration of each interview was between 32 and 44 min. The purpose of the interviews was to explore the topics arising in the focus groups in more depth, and to capture the views of individuals who were unable or unwilling to participate in the group setting (for example, those for whom health was a barrier to attending).

A purposive, maximal variation sample was selected from the database of 188 households currently participating in the Smartline project. Using the project's baseline data to inform sampling, the aim was to include male and female participants of a range of ages and education levels, with various levels of experience with technology, living in different areas within the social housing setting (Camborne, Pool, Illogan, and Redruth). Inclusion criteria were adults aged 18 years and older with mental capacity to participate. Potential participants were invited to take part in either focus groups or interviews via post or e-mail, with detailed written information on the study provided. Those interested in participating returned a reply slip via post or e-mail, and were then contacted by the researchers with details of the focus group time and location or to arrange a suitable time for an interview. Signed consent forms were completed by all focus group and interview participants. Participation did not involve incentives; no payments were made to participants, and no devices were provided.

### Data collection

Theory-and-evidence-based topic guides were produced for the focus groups and interviews, based on findings from a literature review conducted by the study team. For example, the Technology Acceptance Model^23^ and barriers and facilitators to technology use in similar populations (such as older, rural-dwelling adults)^24,25^ were incorporated into the topic guide as discussion prompts. The outline topic guide is presented in Table 1. The findings of the review were also used to identify eight different types of technology (potential digital interventions) that had been shown to be feasible in similar settings in previous studies. The eight digital technologies are presented in Table 2.

**Table 1.** Focus groups topic guide.

Topic	Duration
1. Welcome of participants and overview of aims	5 min
2. Experiences with digital technology, including: Existing technology use Digital competence Barriers and facilitators to technology use Willingness to use new digital technologies	25 min
3. Feasibility and acceptability of digital technologies, including: Introduction of fictional characters Perceived usefulness, ease of use and interest in eight different digital technologies (presented in turn)	45 min
4. Conclusion and acknowledgement	5 min

**Table 2.** Eight types of technology (potential digital interventions) discussed in focus groups and interviews.

A Wearable activity monitor (e.g. Fitbit^®^)
B Social messaging or networking (e.g. WhatsApp or Facebook group)
C Smartphone app (e.g. walking or home-based exercises)
D Social online gaming (e.g. poker, Scrabble, puzzles)
E Video calls (e.g. Skype)
F Virtual assistant (e.g. Amazon Alexa)
G Digital soundscapes (e.g. music, sounds of nature)
H Electronic books and audiobooks (e.g. BorrowBox Application)

The focus groups were co-facilitated by two experienced researchers (SAB and TW) with support from additional members of the study team. The focus groups involved in-depth discussion of the key topics (see Table 1), and participants also completed response sheets during the sessions. The response sheets included a self-rating of each participant's digital competence on a scale of 1 to 9 using the UK Government Digital Inclusion Scale^26^ and a preference ranking task for the eight candidate digital technologies for use in interventions (Table 2). Prior to preference ranking, each type of technology and its potential use for health and wellbeing was explained in turn using visual prompts (laminated A4 images and descriptions, see Additional File 1). This process helped to reduce the influence of familiarity on participants’ perceptions of acceptability. The preference ranking was completed individually following the group discussion; participants placed the technologies in order of preference based on perceived usefulness for health and wellbeing, perceived ease of use, and personal level of interest.

Because some of the discussion topics (such as social isolation and loneliness) were sensitive issues, the focus group participants were given the option to talk about two hypothetical characters (one socially isolated and the other physically inactive) rather than themselves. The characters were based on archetypes created from data based on the entire Smartline cohort.^27^ The characters and descriptions as presented to participants are shown in **Additional File 2**.

The telephone interviews were conducted by a single researcher (SAB). The topics and order of discussion were the same as in the focus groups, but interviewees rated their perceived digital competence prior to the interview (this was returned with the consent form). Interviewees did not rank the potential interventions as in the focus groups but instead talked about the perceived acceptability of each type of technology following a description by the researcher (using the same prompts as the focus groups).

All interviews and focus groups were audio-recorded (with the participants’ consent) and transcribed verbatim by a professional transcription company, who were informed of the need to maintain confidentiality and anonymity. All participant identifiable information was removed from the transcripts.

### Data analysis

All focus group and interview transcripts were organised and coded using NVivo 12 software.^28^ Thematic analysis, which is compatible with the interpretivist paradigm, was used to identify patterns or common themes within the data. The guidance of Braun and Clarke was closely followed during this process.^29^ Following familiarisation with the data, two researchers (SAB and TW) independently coded the transcripts before meeting to compare responses and to agree on the coding scheme. There was high agreement between the researchers’ coding; with minor discrepancies resolved through discussion. The coding scheme was subsequently verified by an independent researcher (KM). Both inductive and deductive analysis were used in coding; the former identified themes arising from the data, while the latter drew on the theory-based categories and concepts within the topic guides (e.g. perceived usability and ease of use from the Technology Acceptance Model^23^).

Data on participants’ self-rated digital competence and preference ranking of the digital interventions (i.e. quantitative data) were analysed descriptively. The range and mean score on the Digital Inclusion Scale was calculated for the study sample. For the preference ranking task, a score of 8 was assigned for each participant's preferred type of technology, 7 for the second preferred and so on (with a score of 1 for the least preferred technology). The individual ratings were then summed to produce an overall score for all of the focus group participants. These data are discussed next and considered together with the qualitative data to produce a comprehensive picture of the findings.

## Results

Details of participants’ demographics and characteristics are provided in Table 3. The mean age of participants was 64.3 ± 12.7 years, and the majority were of white ethnicity (16/23, 70%), educated to lower secondary level (13/23, 57%), and a large proportion were retired (10/23, 43%). Participants’ self-rated digital competence ranged from 3 (willing and unable) to 9 (expert), with a mean rating of 6.4 (task specific). Four main themes identified from the interviews and focus groups were: experiences and perceptions of digital technology; barriers and facilitators to technology use; perceived benefits and negatives; and training and support.

**Table 3.** Demographics and characteristics of focus group and interview participants.

Variable	Participated in study (*n* = 23)
Age in years	
Mean (SD)	64.3 ± 12.7
Range	36 to 80
Gender, *n* (%)	
Male	10 (43%)
Female	13 (57%)
Ethnicity, *n* (%)	
White	16 (70%)
Other	2 (9%)
Unknown	5 (22%)
Highest level of education, *n* (%)	
Lower secondary school (11–16 years)	13 (57%)
Upper secondary school (16–18 years)	3 (13%)
University / college degree	3 (13%)
Unknown	4 (17%)
Employment status, *n* (%)	
Employed (full- or part-time)	5 (22%)
Student / training	2 (9%)
Long-term sickness / disability	3 (13%)
Retired	10 (43%)
Unknown	3 (13%)
Self-rated digital skills (using the UK Government Digital Inclusion Scale), *n* (%)	
1 Never have, never will	0 (0%)
2 Was online, but no longer	0 (0%)
3 Willing and unable	3 (13%)
4 Reluctantly online	0 (0%)
5 Learning the ropes	4 (17%)
6 Task specific	2 (9%)
7 Basic digital skills	8 (35%)
8 Confident	5 (22%)
9 Expert	1 (4%)
Mean rating (SD)	6.4 ± 1.7

Note: SD = Standard Deviation.

### Experiences and perceptions of digital technology

Existing levels of experience with digital technology varied widely. Some individuals reported only having used a basic mobile phone for calls and texts, while others were frequent users of multiple types of technology, including smartphones, tablets, laptops and smart televisions. Participants used technology for a range of purposes, including communicating with family and friends via e-mail or video calls, socialising (e.g. online forums), finding information, online shopping and banking, and online gaming.

Despite using technology for specific tasks, there was some confusion over various technologies and terminology. Many participants showed a lack of awareness and understanding of different technologies and how these could benefit them:

“*I don't know, to be honest I don't know, like I say, I’m the least techno-savvy person. I don't know what's out on the market, what is, what ain't, what can be done, what can't be done.”*

(P4, focus group 3)

Nevertheless, technology was generally perceived in a positive light, even by those with little experience:

“*Technology is a marvellous thing. I’m not knocking it because I don't understand hardly any of it… It's marvellous.”*

(P2, focus group 1)

The majority of individuals were willing and keen to learn and try new types of technology. For example:

“*I’ll give anything a go. I’m great at trying!”*

(P2, focus group 1)

### Barriers and facilitators to technology use

**Figure 1.**
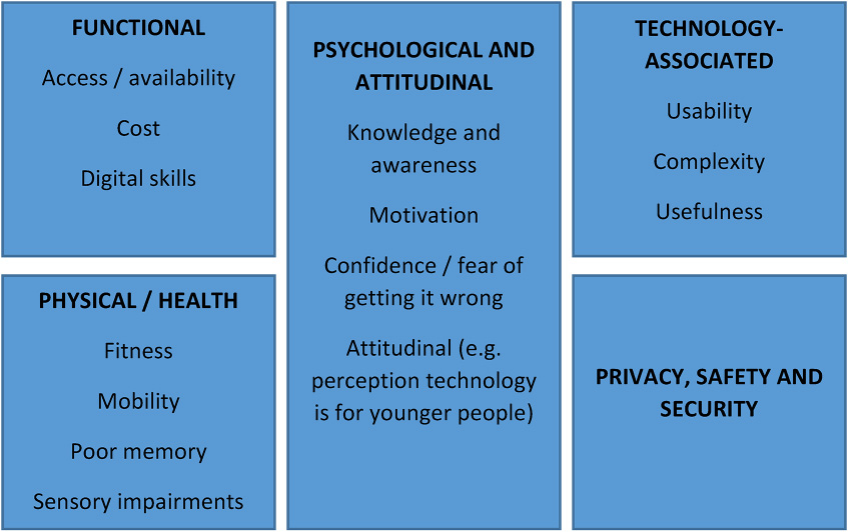
Factors influencing technology use in a social housing population (based on focus groups and interviews)

Participants reported experiencing a number of barriers that prevented or discouraged them from using technology, and related factors that encouraged them to use technology more. Based on inductive data from the focus groups and interviews, and guided by the work of Neves et al. and Batsis et al.,^24,25^ these factors were classed into five categories of barriers and facilitators (Figure 1).

#### Functional

Functional barriers and facilitators included availability of technology and access to the internet. Some participants did not have an internet connection and others reported their connection was too slow. Cost was an important factor for many:

“*I haven't got a smartphone, unfortunately. A bit too expensive for me.”*

(P3, focus group 3)

“*All this new tech comes out at silly prices.”*

(Interview P3)

One individual shared a neighbour's internet connection, while another used the local library computer facilities to overcome these issues.

Several participants felt they lacked the necessary digital competence to use technology effectively. One of the interviewees felt that her lack of experience and skills was holding her back from trying new technologies:

“*There's new technologies which are out, but I think I would hesitate to use them, because there's always…* *I’ll make a mistake, or I wouldn't know how to.”*

(Interview P4)

#### Physical / health

Physical and health issues influenced technology use for a few, typically older, participants. These included fitness and mobility issues (particularly in relation to technology such as activity monitors). However, poor memory and sensory impairments also impacted on participants’ functional capacity to use the technology. For example:

“*My memory isn't quite…* *Yes, I can be told and learn from it, but then trying to remember it. I would try, but… truthfully I wouldn't remember some of it.”*

(Interview P4)

P5 “My sight is not brilliant and sometimes I have to really look and think, is that legit? Is that not? Do you know what I mean? I’m sure I’m not the only one.”

P2 “Do I press that button or not?”

(Focus group 1)

#### Psychological and attitudinal

Psychological barriers (and associated facilitators) to technology use included knowledge and awareness of the different types of technologies and their potential uses, motivation to use the technology and confidence. Several participants reported low confidence, and some recounted bad past experiences which had led to a fear of ‘getting it wrong’ or ‘messing it up’:

“*I tried to do a computer course over at [college] years ago and I buggered up their computer real proper. He said just press a few keys, nothing will happen. So I did and it didn't work again.”*

(P4, focus group 2)

There was evidence of some attitudinal barriers, for example perceptions that technology is for younger people or too difficult to learn at an older age:

P5 “As you get older, you sometimes miss what to… Younger people, they pick up on things a lot more quickly, don't they?”

P4 “Yes, easy.”

(Focus group 1)

#### Technology-associated barriers / facilitators

The usability and perceived usefulness were key factors that determined the extent to which participants used different digital technologies. Participants preferred technologies that were perceived to be user-friendly, easy to use and accessible, and were more willing to persevere with technology that ‘does what it says on the tin’:

“*Anything that's easily accessible… the ease of use, which I think would be a great advantage without anything else… And if it’ll do what it says on the tin and do it, then I’m quite happy with sitting there for an hour and go, oh, that's wrong, I’ll try that. If I know that, at the end of that, I’m going to achieve what I set out to do.”*

(P2, focus group 1)

There was a general consensus that technology should have a clear purpose or health and wellbeing benefit for the user:

P4 “I mean, if it was technology that could physically help me then I’d be interested, but having technology for technology's sake, just because it's on the market…”

P5 “Yes, I agree.”

P4 “I mean, if it's useful to me and it can help me throughout my days, then…”

P1 “I’m all about technology to help rather than technology for technology's sake.”

(Focus group 3)

#### Privacy, safety and security

The issues of privacy and safety, particularly in relation to online shopping and banking, arose without prompting in each of the focus groups. Many of the participants were very concerned about online fraud, several personally knew people who had been affected and some had themselves been victims of scams. This had resulted in mistrust of the internet and was a major barrier to doing things online:

“*In the past I’ve been burnt on it so I don't ever do anything online. Don't buy online. I don't do banking online.”*

(P1, focus group 1)

There were also some concerns over sharing of personal information online. The information requested by some smartphone apps was seen as intrusive, and there was some anxiety related to the recording of data by technologies such as virtual assistants:

“*I just found out recently that… the Alexa stuff… these machines can actually hear what you’re saying, so it's really like Big Brother's watching you with a big camera up there. It definitely worries me… that seems to be really intrusive.”*

(P1, focus group 2)

This barrier was related to a lack of confidence and perceived digital competence; several participants did not feel that they had the ability to distinguish between legitimate websites, e-mails and apps and those that were not. How to navigate security and verification processes were not understood by some and this presented a barrier to online transactions.

### Digital technology for health and wellbeing

#### Views on technology for health and wellbeing

Despite the lack of knowledge and awareness of different types of technologies reported by many participants, there was an agreement across the focus groups and interviews that technology has many potential benefits for health and wellbeing. The main perceived benefits included the promotion of a healthy lifestyle, reducing isolation and loneliness through online communications, and accessing health information.

The potential negative impacts of technology were also recognised. These included addiction (in particular to online gaming and gambling), privacy and security concerns, and over-reliance or dependence on technology which could lead to physical inactivity and laziness. Some participants talked about the loss of in-person interaction and the resulting decline in interpersonal skills:

P4 “Too many skills are being lost. Communication skills are being lost, letter writing skills are being lost. I actually hate people that sit in a room together and, rather than talk to each other, they text each other… I see it more as a barrier to communication.”

P1 “It's the social aspect as well. We’re losing that.”

(Focus group 3)

#### Ranking task: preferred types of technology

As part of the focus groups and interviews participants were asked to rank their preferred technology based on eight identified digital technologies (see Table 2). Although the exercise found individual differences in the specific types of technology preferred by participants, all eight technologies (Table 2) were perceived as feasible, acceptable and potentially useful for helping them or others in their community to be healthier and happier. The results of the preference ranking task are presented in Table 4.

**Table 4.** Preferred types of technology (potential digital interventions) for health and wellbeing according to preference ranking task in focus groups.

Rank	Type of technology	Total score
1	A Wearable activity monitor (e.g. Fitbit^®^)	75
2	F Virtual assistant (e.g. Amazon Alexa)	63
3	B Social messaging or networking (e.g. WhatsApp or Facebook group)	59
4	H Electronic books and audiobooks (e.g. BorrowBox)	56
5	G Digital soundscapes (e.g. music, sounds of nature)	45
6	E Video calls (e.g. Skype)	43
7	C Smartphone app (e.g. walking or home-based exercises)	35
8	D Social online gaming (e.g. poker, Scrabble, puzzles)	30

Note: Participants ranked the technologies according to perceived usefulness for health and wellbeing, perceived ease of use, and personal level of interest. Each participant's preferred type of technology was given a score of 8, second preferred 7 etc. Individual scores for each technology were summed for all focus group participants.

Wearable activity monitors were preferred overall; these devices were perceived as easy to use and useful for monitoring various aspects of health (physical activity, sleep and heart rate) and for providing motivation to change behaviour. Some individuals expressed that the devices could be used to help them to achieve goals set by their doctor, such as monitoring their heart rate and sleep. A few participants were already using this type of technology, and those that were not showed a keen interest to try it:

“*Wow. That's the sort of thing I’m interested in. Something that's useful.”*

(P1, focus group 3)

While perceived acceptability of activity monitors was high for most participants, physical health was a barrier for some:

“*I can't do a lot of exercise, just based on my medical [condition], unfortunately”.*

(P3, focus group 3)

Virtual assistants were rated second; the wide range of functions of these devices and multiple ways to improve health and wellbeing were recognised, including providing health information, communicating with others (i.e. increasing social connectivity), assisting with physical tasks (e.g. controlling electrical devices) and providing entertainment. Of all the technologies, virtual assistants were perceived as easy to set up and use. These devices were seen as particularly useful for people with physical health conditions and disabilities, or isolated elderly people:

“*I think it would be a great help for the older people who are on their own in the house. Because you can ask it to turn the TV on or the lights on or music, which maybe they can't do themselves. It would help benefit them.”*

(Interview P4)

Social messaging (such as WhatsApp) was generally preferred to social networking sites such as Facebook. Several participants used social messaging to keep in touch with friends and family:

“*I’ve got WhatsApp. That's perfect as well… because, I can contact the grandchildren, and we can send videos…”*

(Interview P4)

One elderly, housebound participant reported that his friends used social messaging as a monitoring system, to ‘check in’ with him on a daily basis.

“*Because I live on my own I have friends that I text. Well, it's message, actually, on WhatsApp because it's free. So every morning I message them and they message back. That means I’m still alive and I’m okay. If I don't message, within ten minutes they’re knocking on my door.”*

(Interview P2)

There was high interest in social messaging groups for people with shared interests, or community or befriending groups for those who are isolated:

“*If they came up with an app for people that spend time on their own, and they just need someone to chat to… When you’re so vulnerable…* *you’re on your own. Pensioners. Even people my age suffer with it. Just that you could reach out to someone like-minded… to talk to someone who understands what you’ve been through or what you’re going through. It would be nice if there was a group like that.”*

(P1, focus group 3)

### Training and support

All of the participants, with the exception of the one ‘expert’ user, felt that they needed further information, training and support, particularly in relation to setting up and using new technologies. Existing sources of support included online information, user guides, and help from family and friends. User guides (for example, for smartphones) were typically viewed as complex and unhelpful:

“*They give you a little instruction handbook which is absolutely in gibberish. You can't really understand anything from it. You need someone to tell you in simple terms how to use it.”*

(P3, focus group 2)

Support was often provided by younger family members, usually children and grandchildren:

“*My daughter is spot on with this sort of stuff. She does everything for me so that's how I know quite a bit, but it's all done for me, like my computer's set up and that, so that's it.”*

(P1, focus group 2)

Family members also helped participants with the sign-in process, managing e-mail and online accounts, and with security concerns:

“*These scam things… If I see something, I ring my daughter and say, ‘is that a scam or is that true?’”*

(P3, focus group 1)

However, family members were often too busy to help, and participants expressed a wish for a more patient trainer who would not patronise them:

“*My son… won't let me on it [the internet]. He won't tell me how to use it either. Because he says he does that all day, he's not doing it when he's home.”*

(P4, focus group 2)

“*My lot are… 30 to 40 and they are, ‘Oh, Mother, you’re not stupid. You can do it.’ And I think, I’m not stupid… but they make you feel stupid.”*

(P3, focus group 1)

Some participants expressed a wish for one-to-one support, while others said they would prefer group training so they could learn from others in a similar situation. There was a strong belief that technology should not be used to replace in-person interaction, and the importance of maintaining the ‘human’ element in digital technologies and training was emphasised.

“*I’d rather meet somebody and talk to them. Actually I don't use these talks, social media business. Because to me they should ban all that so people actually talk to each other face to face.”*

(Interview P2)

“*[I prefer] face to face, just in person… it's what we are, that's how we evolved.”*

(Interview P3)

## Discussion

Digital technology has the potential to improve health and wellbeing in the home and community. Dozens of digital health projects have been conducted in Europe in parallel with the proliferation of mobile technologies for improving health and wellbeing.^30^ However, much emphasis has been placed on the effectiveness of these projects, typically assessed using quantitative measures, at the expense of qualitative methods that capture important aspects such as feasibility and acceptability.^8^ This is despite a growing literature that emphasises the need for an increased understanding of barriers, enablers, attitudes and behaviours to digital technology for health and wellbeing.^31^ In response to this need, this scoping study explored the feasibility and acceptability of digital technology for health and wellbeing in a social housing cohort living in a low income, geographically isolated, rural area.

The study found positive attitudes to digital technology, a willingness to trial new technologies, and a clear need for training and support in technology use. The findings add support to the Technology Acceptance Model, which proposes that the acceptability of new technologies is largely determined by their perceived ease of use and perceived usefulness,^23^ and also influenced by external variables such as social influence.^32^ We found that these factors were key in participants’ existing use of technology and perceived acceptability of new technologies. There was a strong consensus that technologies should be easy to use and have a clear purpose, such as making life easier or improving health and wellbeing, rather than ‘technology for technology's sake’. Social influence was an important factor with the majority of participants seeking help from family and friends. This source of support has previously been reported in studies of older adults.^24,33^ However, it should not be assumed that family and friends have the skills, time, and willingness to support. Indeed, importantly, participants had a strong desire for independent support that would allow them to be less dependent on family and friends. External, one-to-one training was seen as essential to help to realise the usefulness of digital technology.

Regarding barriers and facilitators to technology use, the findings support and extend those of existing studies. Neves and colleagues (2013) reported three categories of barriers to technology use in adults aged over 64 years – functional, physical and attitudinal.^24^ We identified five main categories of factors influencing technology use in our social housing cohort with mainly older, but some younger adults: functional, physical / health, psychological and attitudinal, technology-associated barriers, and privacy, safety and security.

Specifically, building on the work of Neves et al.,^24^ we found evidence for functional barriers (access and availability of technology, and digital competence), we extended the physical barriers category to include health barriers (fitness, mobility, memory and sensory impairments), and incorporated attitudinal barriers into a wider ‘psychological’ category that included knowledge and awareness, motivation and confidence. Within the psychological category, fear of new technology (sometimes based on past experience) was an issue for several participants. Previous studies have also found a need to overcome a fear of new technology before learning to use it effectively.^34^

Based on our findings, we also proposed two additional categories of factors influencing technology use – technology-associated barriers and facilitators (usability, complexity and usefulness) and privacy, safety and security. Concerns over privacy were a recurring theme and a key inhibiting factor in technology use; privacy has previously been reported as a barrier to technology use in rural-dwelling older adults.^25^ Perceptions and experiences of online privacy, safety and security issues require further qualitative research, particularly amongst vulnerable groups, and should be a central consideration in the design of digital interventions.

Many participants had low digital confidence and self-rated competence, and lacked knowledge and awareness of which technologies are available and their capabilities for improving health and wellbeing. Although most of the participants in this study sought support from family and friends in using technology, this was often not enough, and there was a strong desire for further support, training and guidance. Previous research with older adults found that increasing contact and familiarity with technology (computers) resulted in a number of beneficial outcomes, including more positive attitudes towards technology and improved self-confidence.^35^ The human element of digital technologies and support with using technologies was perceived as extremely important. This finding has also been reported in studies of adults with chronic health conditions; for example, older adults with chronic pain have shown a preference for digital healthcare interventions to be delivered alongside in-person visits.^36^

The generalisability of the findings relates to the participant population; predominately older individuals living in social housing. For this group, a key finding is that acceptability is primarily influenced by ease of use and the technology's functionality to achieve personal and meaningful health goals; these may include maintaining and improving health conditions. Our findings show that this group prefer technologies which have multiple functions, and which may improve health and wellbeing in a number of different ways; these might extend beyond the primary marketed function of the technology. For example:

- Wearable activity monitors to encourage self-monitoring of physical activity, sleep and heart rate; enable goal-setting; and provide motivation for behaviour change.
- Virtual assistants to facilitate access to online health information or services; provide assistance with physical tasks (particularly important for people with disabilities); and provide entertainment.
- Social messaging (e.g. apps) to connect with family, friends or the local community, thereby reducing isolation and loneliness. This may also be used as a ‘monitoring’ system for checking in with elderly individuals who live alone.

Most importantly, this study provides a strong foundation for the co-creation of digital interventions. The thorough initial needs assessment has already been used to inform and shape interventions in the Smartline project. This includes a ‘getting online, staying connected’ project in collaboration with the Digital Inclusion team in the local Council, an educational intervention comprising one-to-one support in using digital technology, which is currently being trialled. Future interventions based on the preferred digital technologies identified in the focus groups and interviews (i.e. wearable activity monitors, virtual assistants and social messaging and networking) are being planned for the Smartline cohort and the wider social housing community.

Digital technologies such as health apps and wearables are known to be associated with high attrition and declining use over time.^37^ Studies examining the potential of technologies need to consider uptake but also sustained usage and engagement of the technology. As such, “effective engagement” the level of engagement needed to achieve optimal benefit, may be more important than usage per se.^38^ The potential for effective engagement as well as usage should be considered when piloting technologies in similar populations. Our findings suggest that social influence is an important influencing factor for sustained and effective usage for this population. We posit that if family and friends are also engaged these technologies it will help to achieve sustained engagement. Based on the findings of the present study, we anticipate that engagement is likely to be high, as the participants were willing and motivated to use technologies that they perceive as beneficial to their health and wellbeing.

### Strengths and limitations

A key strength and contribution of this study is the inclusion of social housing tenants, a group that has been under-studied in comparison with clinical populations^39,40^ and other community populations such as elderly adults,^33,41,42^ but that is at disproportionately high risk of digital exclusion.^9,13^ The qualitative methods used enabled an in-depth understanding of the context and needs of this population and allowed a detailed exploration of the potential acceptability of digital technologies. These aspects are often overshadowed by quantitative studies of impact and effectiveness.^8^

One limitation of the present study is the use of telephone interviews rather than face-to-face interviews. This method was selected as it was perceived to be more convenient and less invasive for participants, and there is evidence that well-conducted telephone interviews may yield high quality data with as much depth as face-to-face interviews.^43,44^ Another limitation is that the research was conducted with a relatively small sample of participants within the Smartline project. While the researchers tried to ensure a diverse, maximal variation sample, some selection bias may be observed, with individuals who were more interested in technology more likely to participate. Nevertheless, the study still identifies a need for more targeted and accessible support for those with an interest in technology. The findings may differ from other social housing communities in the UK and internationally. Future studies should aim to address these issues.

## Conclusions

The findings of this study indicate feasibility and acceptability of different digital technologies for promoting physical and psychological health and wellbeing, and reducing social isolation in a rural social housing cohort. Overall, we found positive attitudes to technologies that participants perceived as useful to them, a willingness and desire to trial new digital technologies, and a clear need for training and support in technology use. Although there are various barriers to consider, the work provides a strong basis for the co-creation of digital interventions in this group, beginning with a one-to-one support and training programme that aims to improve confidence and competence in using technology. In addition to the contributions to the theory and evidence base, the findings are likely to be of interest to a number of stakeholders, including researchers developing similar digital interventions, technology designers and developers, educational organisations, social housing providers and councils. The study exemplifies the importance of conducting a transparent needs assessment prior to intervention design and technology procurement.

## Declarations

## Supplemental Material

sj-docx-1-dhj-10.1177_20552076221074124 - Supplemental material for The feasibility and acceptability of digital technology for health and wellbeing in social housing residents in Cornwall: A qualitative scoping study Click here for additional data file. Supplemental material, sj-docx-1-dhj-10.1177_20552076221074124 for The feasibility and acceptability of digital technology for health and wellbeing in social housing residents in Cornwall: A qualitative scoping study by Sarah Ann Buckingham, Tim Walker, Karyn Morrissey and in Digital Health

sj-docx-2-dhj-10.1177_20552076221074124 - Supplemental material for The feasibility and acceptability of digital technology for health and wellbeing in social housing residents in Cornwall: A qualitative scoping study Click here for additional data file. Supplemental material, sj-docx-2-dhj-10.1177_20552076221074124 for The feasibility and acceptability of digital technology for health and wellbeing in social housing residents in Cornwall: A qualitative scoping study by Sarah Ann Buckingham, Tim Walker, Karyn Morrissey and in Digital Health
