# Supplementary material for: The feasibility and acceptability of digital technology for health and wellbeing in social housing residents in Cornwall: A qualitative scoping study
Source: Digit Health. 2022 Jan 24;8:20552076221074124. doi: 10.1177/20552076221074124 (PMC8793427; doi:10.1177/20552076221074124)
Supplement: sj-docx-1-dhj-10.1177_20552076221074124 - Supplemental material for The feasibility and acceptability of digital technology for health and wellbeing in social housing residents in Cornwall: A qualitative scoping study [file sj-docx-1-dhj-10.1177_20552076221074124.docx]

**
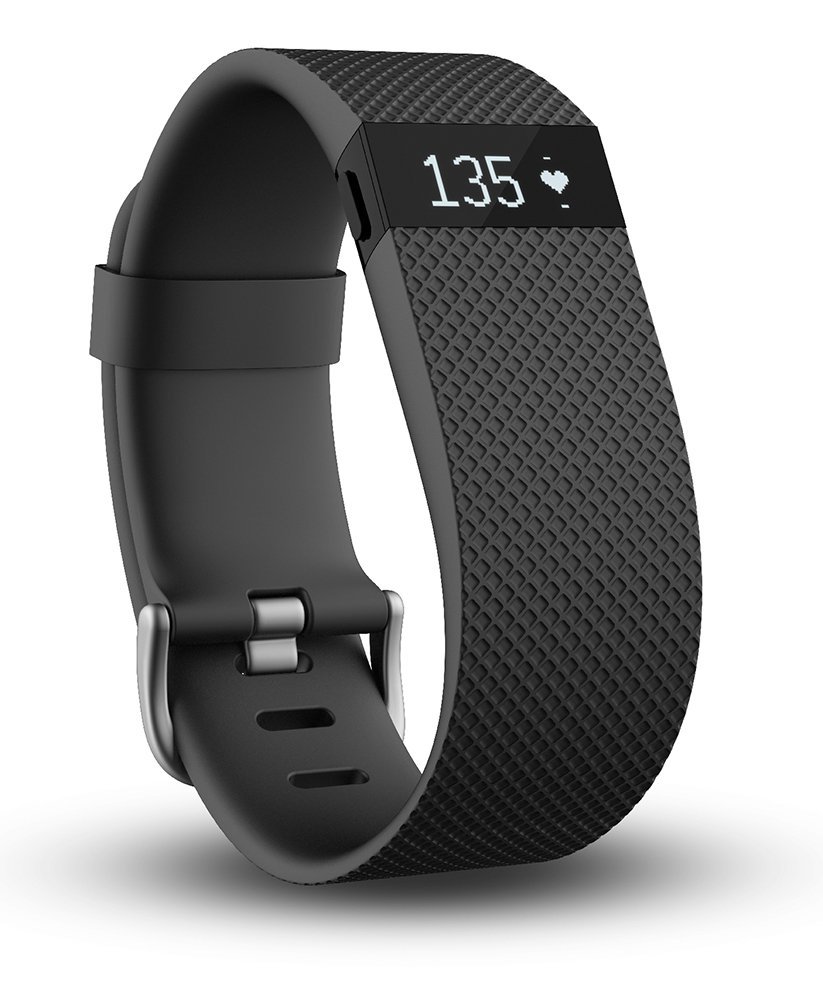

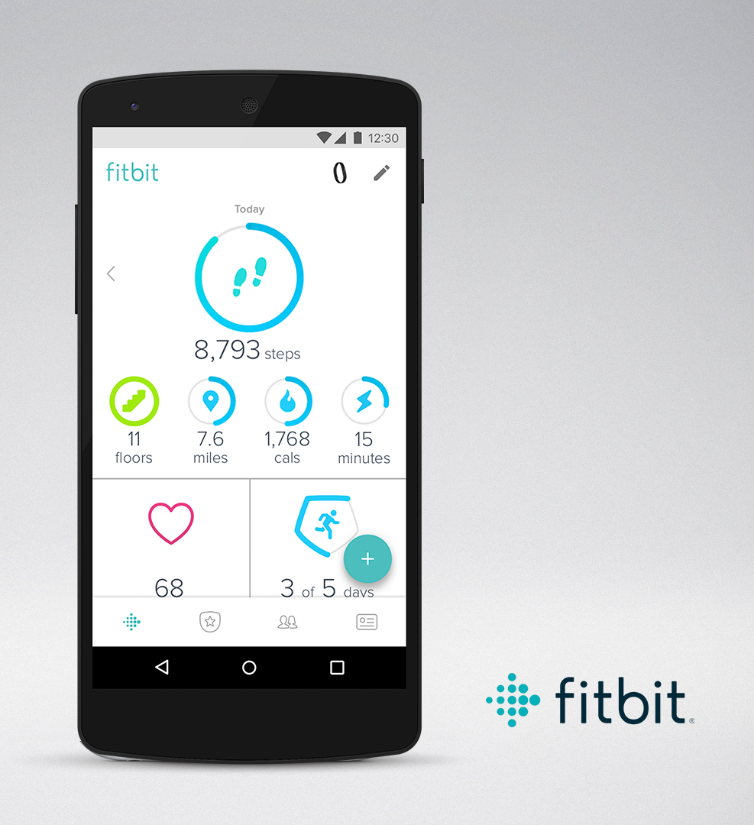
a) Wearable activity monitor (e.g. Fitbit^®^)**

- **Monitor your steps, activity, sleep, heart rate and calories**
- **View data on the screen, smartphone app and/or website**
- **Compare or compete with other people (optional)**
- **Needs to be charged at least once a week**

**
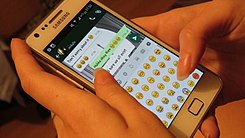
b) Social messaging or networking (e.g. WhatsApp or Facebook group)**


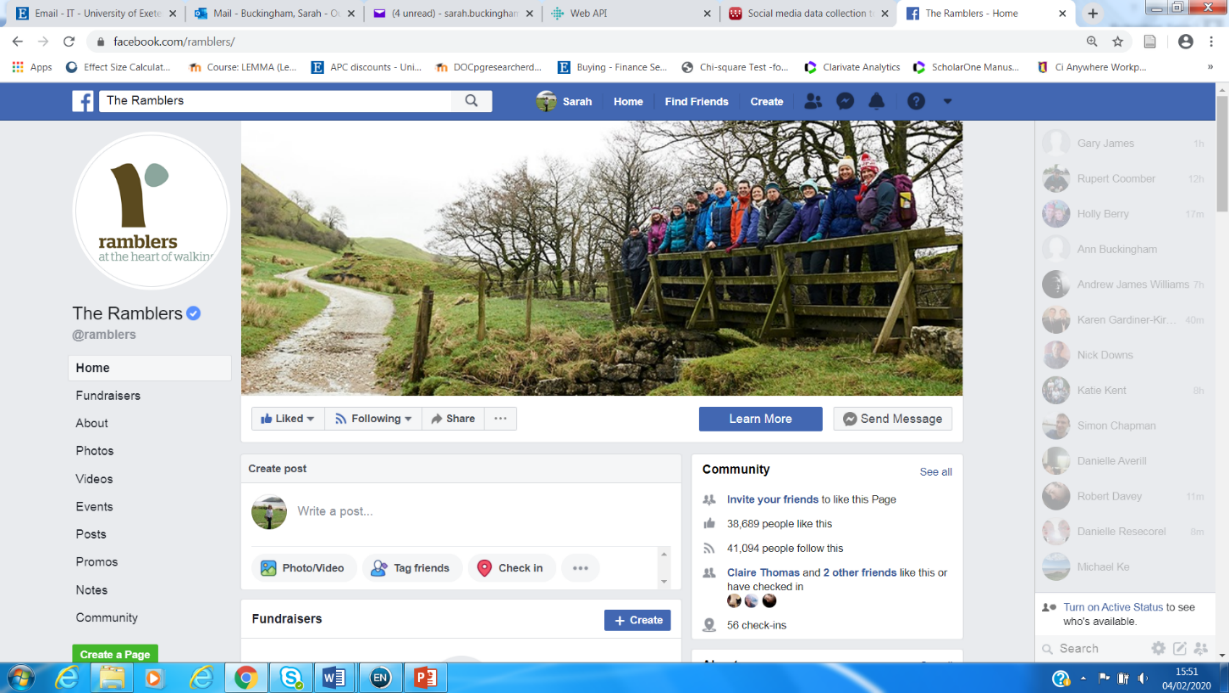


- **Connect a group of people with a shared interest (e.g. walking, reading, local history or community group)**
- **Send group messages, share photos, videos, knowledge and opinions**
- **Could use to organise events (e.g. group outings or book club meetings)**

**c) Smartphone app (e.g. walking or home-based exercises)**

**
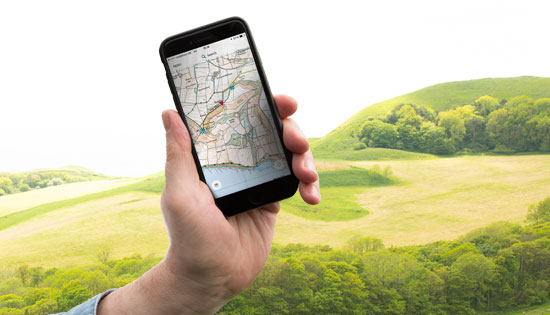
**

**
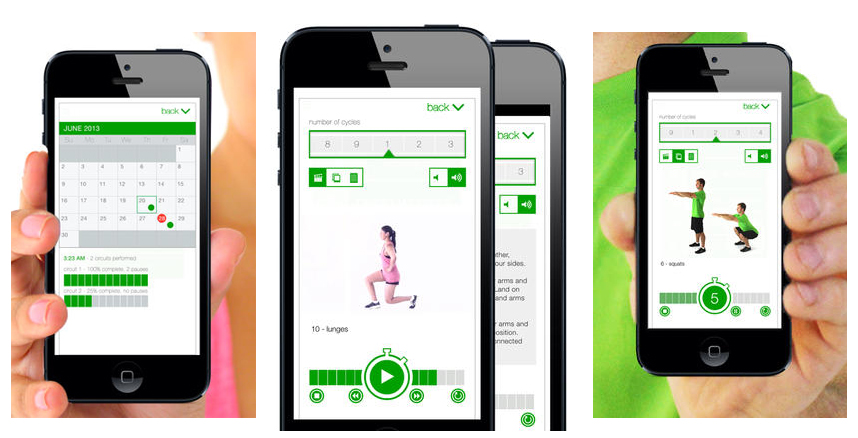
**

- **Use an app on your phone to map walks using GPS (satellite navigation)**
- **Or an app that demonstrates exercises you can do at home and motivates you**

**
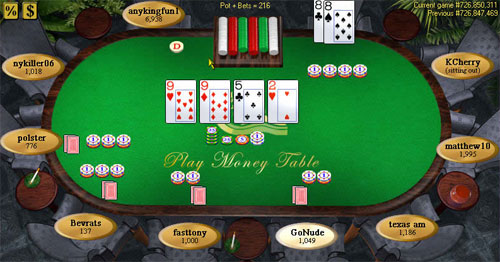
d) Social online gaming (e.g. poker, Scrabble, puzzles)**

**
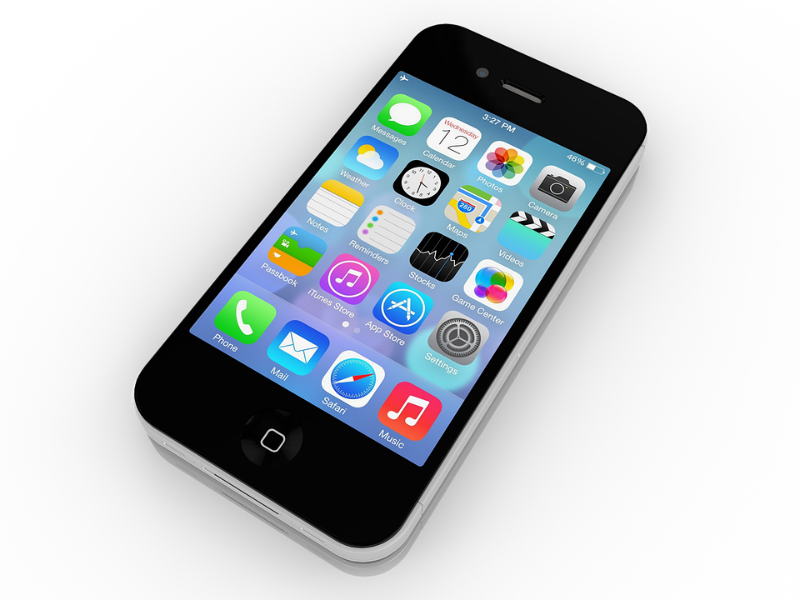
**

**
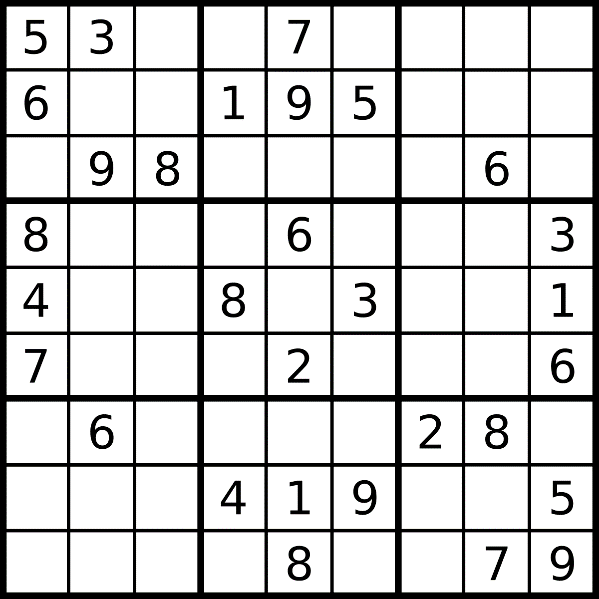

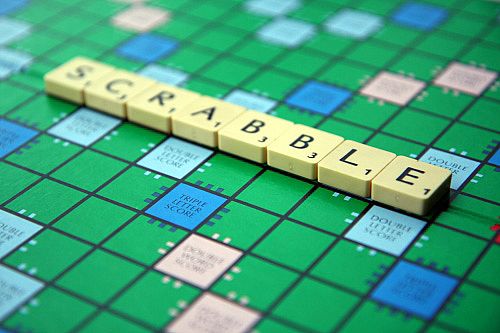
**

- **Play on your smartphone, tablet or computer**
- **Compete with or work with other people to play games / solve problems**
- **Send messages to or directly speak to other players**

**
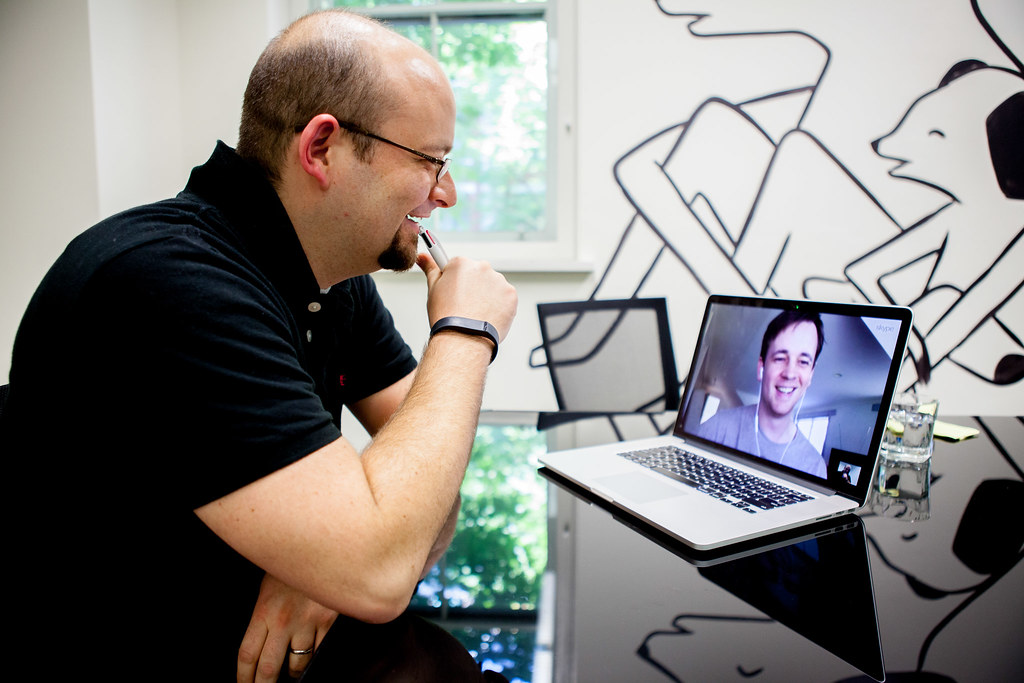
e) Videoconferencing or Skype**

- **Video communication via your smartphone, tablet or computer**
- **Talk to family, friends, volunteers or others in the community**
- **For example, weekly chat with a volunteer**

**
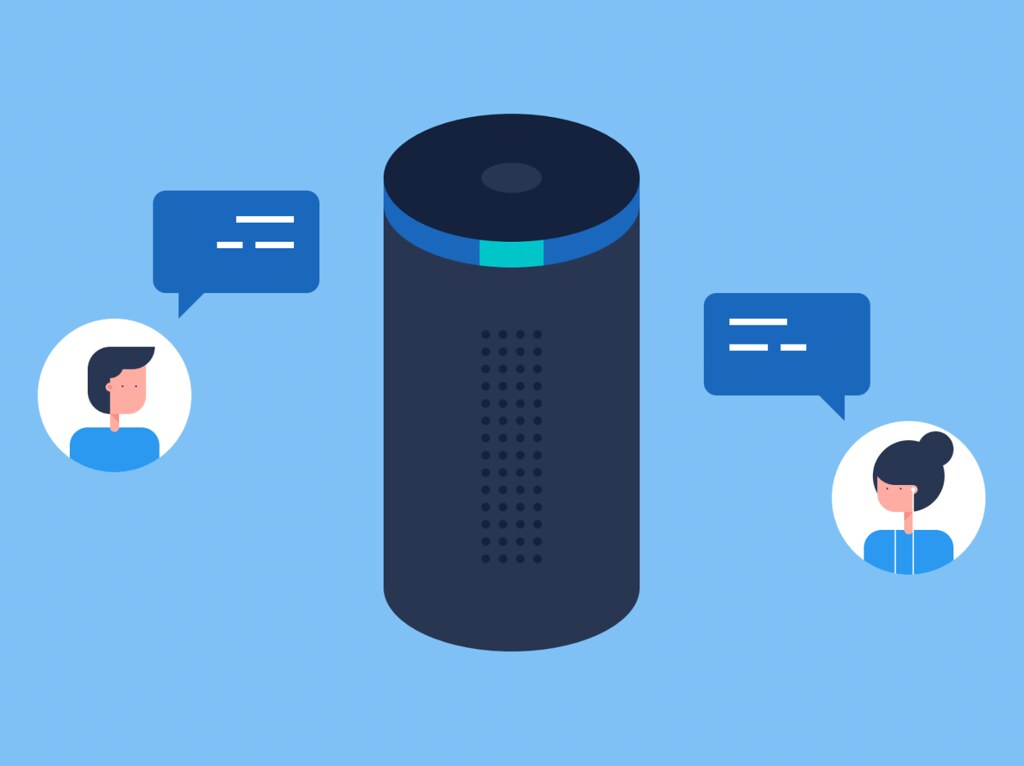
f) Virtual assistant (e.g. Amazon Alexa)**

- **Voice-controlled artificially intelligent device**
- **Can answer questions, provide information (e.g. news, weather and traffic updates), read audio books, play music, set alarms/reminders**
- **Can also control smart devices in the home (e.g. lights, TV, thermostats and locks)**

**g) Soundscapes**

**
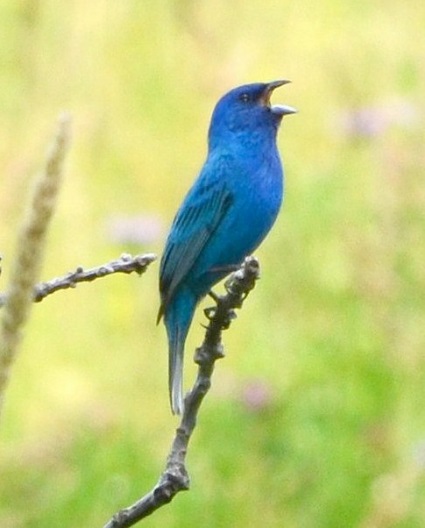

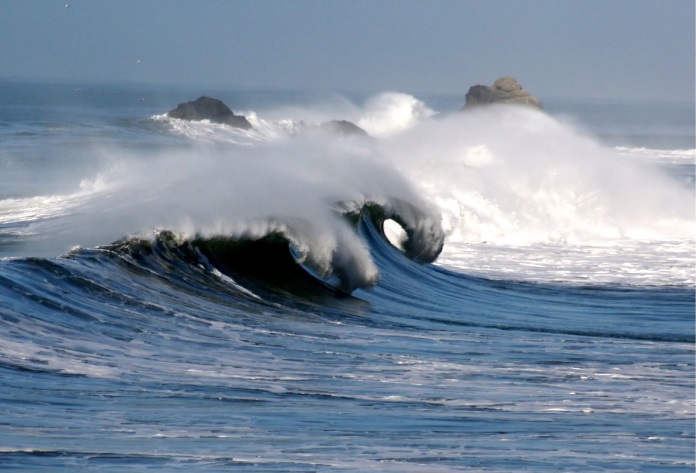
**

**
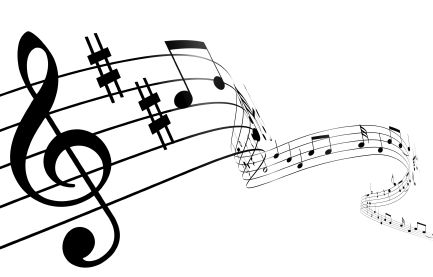

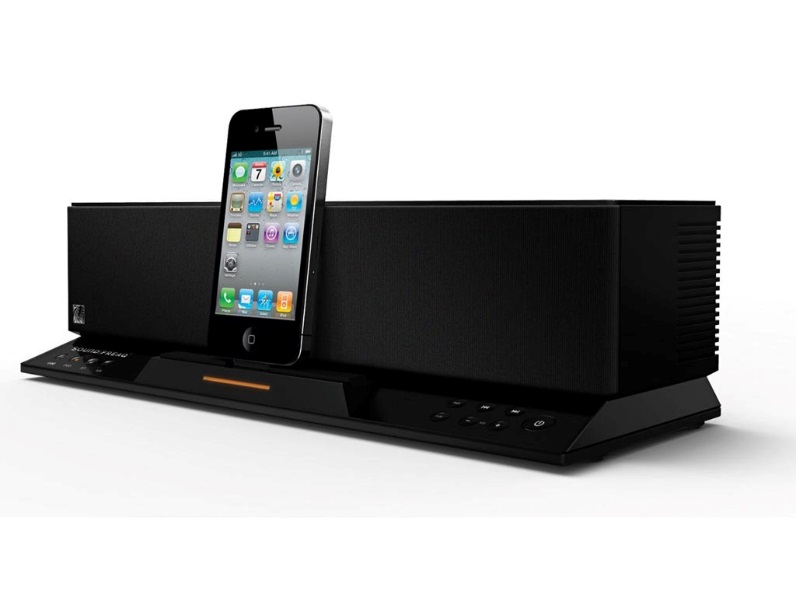
**

- **Play relaxing music or sounds of nature (e.g. birdsong or waves) in your home**
- **Played using a computer, smartphone or tablet**
- **Alternatively, could be played in a shared public space (e.g. common room, green space or park)**

**
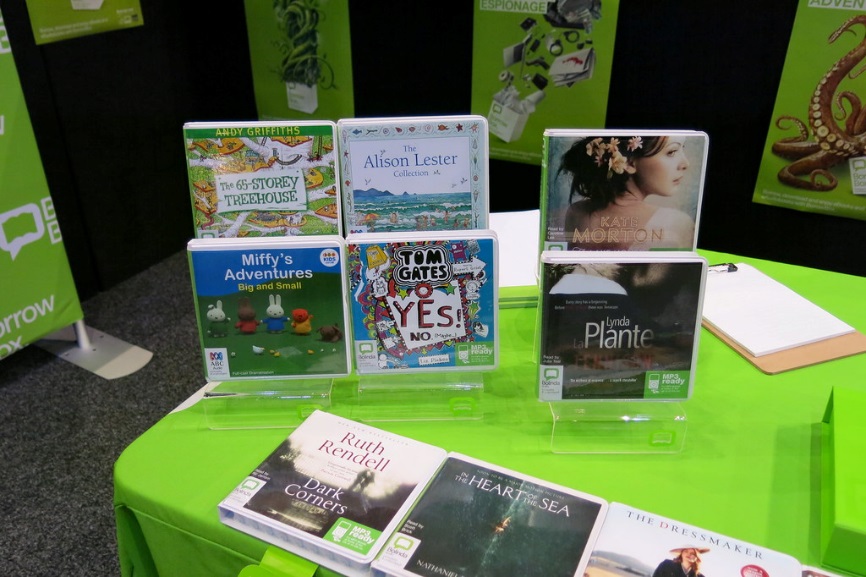
h) Electronic books and audio books (e.g. BorrowBox)**

**
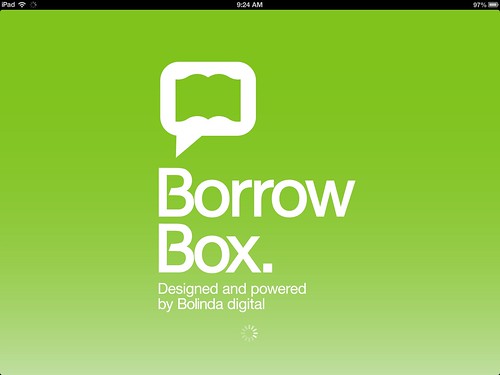
**

- **Borrow eBooks and eAudiobooks free from your local library using your library card**
- **Once downloaded, an internet connection is not required**
- **Read or play through your smartphone, tablet or computer**
