# Supplementary material for: The feasibility and acceptability of digital technology for health and wellbeing in social housing residents in Cornwall: A qualitative scoping study
Source: Digit Health. 2022 Jan 24;8:20552076221074124. doi: 10.1177/20552076221074124 (PMC8793427; doi:10.1177/20552076221074124)
Supplement: sj-docx-2-dhj-10.1177_20552076221074124 - Supplemental material for The feasibility and acceptability of digital technology for health and wellbeing in social housing residents in Cornwall: A qualitative scoping study [file sj-docx-2-dhj-10.1177_20552076221074124.docx]

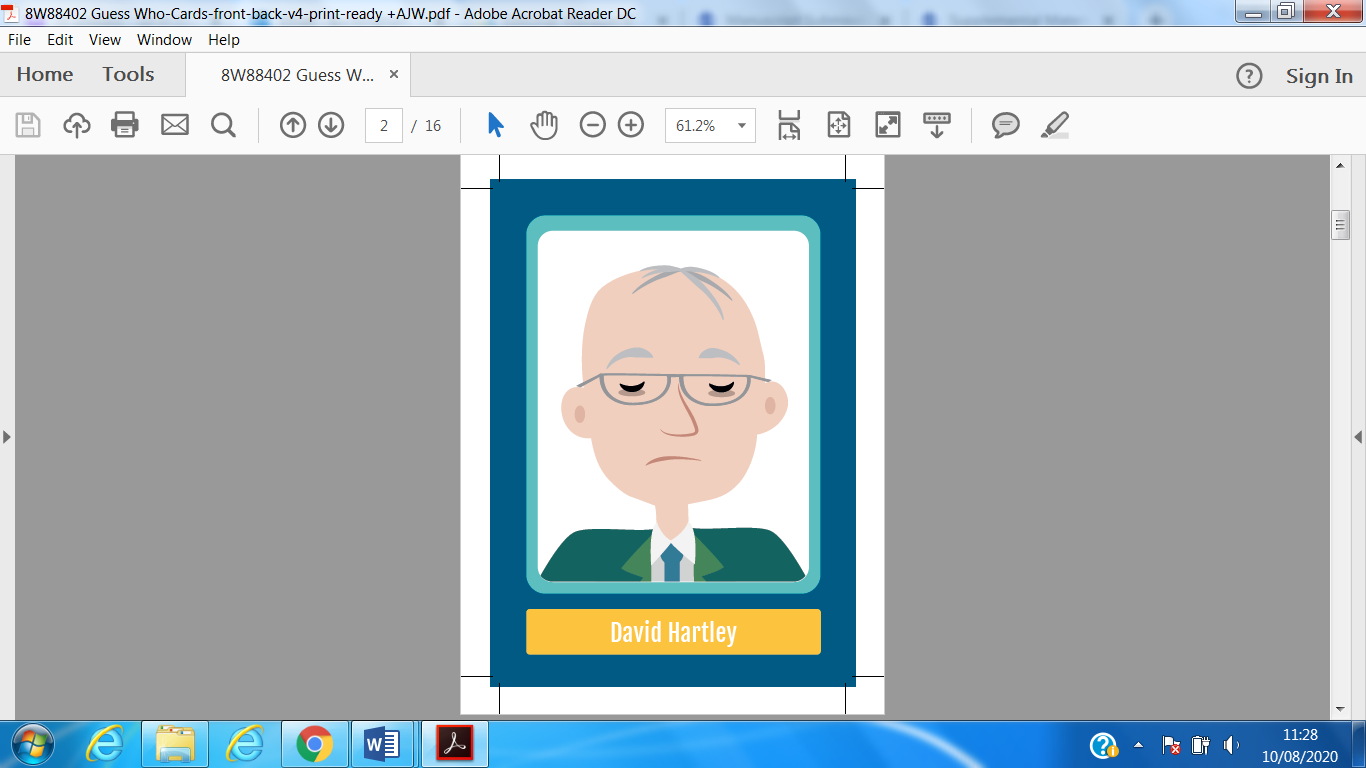
**David Hartley**

*Poor physical health, ex snooker player*

Male, mid-60s, retired. Lives alone and likes where he lives. Attended school to secondary-level. Does not have an internet connection, computer or smart technology. David has arthritis and diabetes which impacts many aspects of his life, including aspirations for exercise, recreation, and learning. Used to play snooker but now spends a lot of time sitting down indoors. He would like to socialise more.


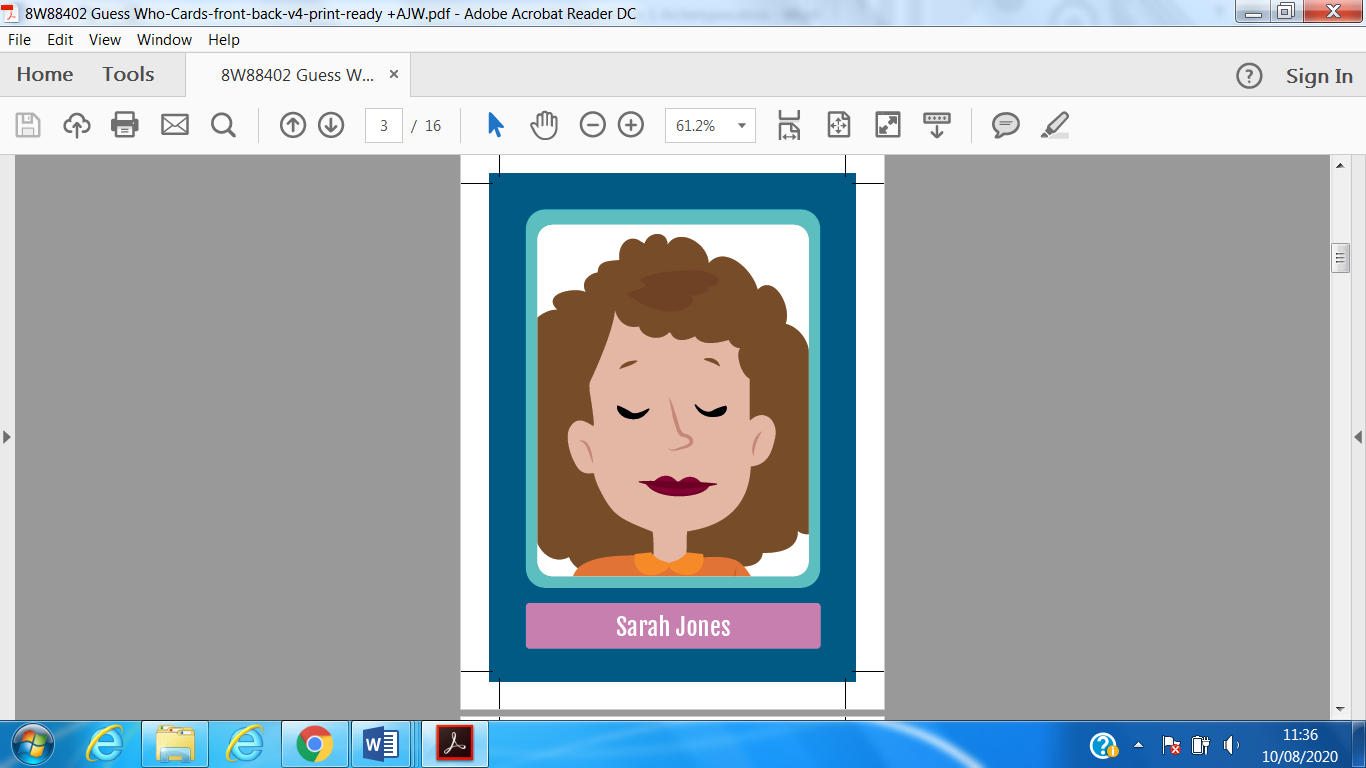


**Sarah Jones**

*Caring responsibilities, active volunteer*

Female, 50 years old. Cares for her two disabled children. Low levels of physical activity. Has a PC, but not internet enabled which can mean she faces difficulties with filling in forms and accessing relevant services. Has a pet cat. Volunteers at the local charity shop and often helps neighbours with their shopping. Wants to be more involved in improving services in the community. Loves to knit when she has time.
